# LDPC-cat codes for low-overhead quantum computing in 2D

**DOI:** 10.1038/s41467-025-56298-8

**Published:** 2025-01-26

**Authors:** Diego Ruiz, Jérémie Guillaud, Anthony Leverrier, Mazyar Mirrahimi, Christophe Vuillot

**Affiliations:** 1 Alice & Bob, 49 Bd du Général Martial Valin, 75015 Paris, France; 2 Laboratoire de Physique de l’École Normale Supérieure, École Normale Supérieure, Centre Automatique et Systèmes, Mines Paris, Université PSL, CNRS, Inria, Paris, France; 3 Inria Paris, 48 rue Barrault, 75013 Paris, France; 4 Université de Lorraine, CNRS, Inria, LORIA, F-54000 Nancy, France

**Keywords:** Qubits, Quantum information

## Abstract

The main obstacle to large scale quantum computing are the errors present in every physical qubit realization. Correcting these errors requires a large number of additional qubits. Two main avenues to reduce this overhead are (i) low-density parity check (LDPC) codes requiring very few additional qubits to correct errors (ii) cat qubits where bit-flip errors are exponentially suppressed by design. In this work, we combine both approaches to obtain an extremely low overhead architecture. Assuming a physical phase-flip error probability *ϵ* ≈ 0.1% per qubit and operation, one hundred logical qubits can be implemented on a 758 cat qubit chip, with a total logical error probability per cycle and per logical qubit *ϵ*_*L*_ ≤ 10^−8^. Our architecture also features two major advantages. First, the hardware implementation of the code can be realised with short-range qubit interactions in 2D and low-weight stabilizers, under constraints similar to those of the popular surface code architecture. Second, we demonstrate how to implement a fault-tolerant universal set of logical gates with an additional layer of routing cat qubits stacked on top of the LDPC layer, while maintaining the local connectivity. Furthermore, our architecture benefits from a high capacity of parallelization for these logical gates.

## Abstract (web summary)

Implementing full quantum error correction incurs a significant hardware overhead. Here, the authors propose a quantum computing architecture combining superconducting cat qubits with 2D local LDPC codes, featuring low overhead and ease of implementation.

## Introduction

The discovery of quantum algorithms capable of solving certain computational problems super-polynomially faster than their classical counterparts has ignited a race to develop quantum computers^1,2^. However, current quantum processors, constrained by quantum decoherence to error rates around 10^−3^ − 10^−4^ per qubit and per operation^3–5^, cannot yet solve practical problems that require 10^7^ − 10^11^ quantum gates^6–8^.

The theory of fault-tolerant quantum computing^9–12^, in principle, offers a solution to this problem. The logical quantum information is encoded with a collection of physical quantum systems using error-correcting codes. Below their fault-tolerance threshold^12,13^, logical qubit errors can be arbitrarily suppressed by increasing the code distance *d* (the minimum number of errors not detected by the code). However, in practice, the implementation of error correction incurs a significant hardware overhead.

For instance, the surface code, despite its high threshold and experimental practicality, demands a significant qubit overhead for the required logical error rate of quantum algorithms^6,14^. This is explained by its relatively poor encoding rate. For a quantum code encoding *k* logical qubits in *n* physical qubits with distance *d*, the encoding rate is defined as the ratio of the number of logical qubits to physical qubits *k*/*n*. The encoding rate of the surface code is given by 1/*d*^2^. For the practical distances of interest falling within the range of 10−30, this leads to a 1% to 0.1% rate. Thus, between 100s and 1000s of physical qubits are needed per logical qubit.

One direction pursued to reduce this overhead is the use of quantum low-density parity-check (qLDPC) codes, which has been the focus of recent intensive research^15^, leading to the discovery of “good” qLDPC codes^16–18^ with a non-vanishing encoding rate and a distance growing linearly with the block size *n*. These theoretical advances have inspired the systematic search and the discovery of small qLDPC codes with higher encoding rates than the surface code^19–21^. However, implementing these superior codes incurs a higher technological cost than the surface code for a fundamental reason^22^. Indeed, Bravyi, Poulin and Terhal showed that the performance of a *local* quantum error correcting code on a 2D lattice is upper bounded by *kd*^2^ = *O*(*n*) (BPT bound)^23^. Because the surface code saturates this bound, improving upon it with a 2D architecture necessarily requires *non-local i.e*. long-range interactions in the processor. While this property is feasible for certain physical platforms like neutral atoms^24,25^, it is more challenging to realize with superconducting circuits, although the biplanarity property of some qLDPC codes may help^20^. Finally, the fault-tolerant construction of a universal set of logical gates, which can be reasonably parallelized on these qLDPC codes is still an active research problem^26–36^.

Another approach to reduce the footprint of the architecture is to optimize the synergy between the error-correcting code and its physical component noise structure^37–42^. In particular, dissipatively stabilized cat qubits^43,44^ stand out among bosonic qubits due to their remarkable property that the bit-flip error rate is exponentially suppressed with the average number of photons in the cat qubit, at the cost of a linear increase in the phase-flip error rate. This drastic scaling difference between these two types of errors leads to qubits with extremely biased noise of $\eta \,\dot{=}\,{p}_{Z}/({p}_{Y}+{p}_{X})\,\gtrsim \,1{0}^{8}$ even for modest numbers of photons, as demonstrated experimentally with a 15 s bit-flip lifetime for a corresponding phase-flip lifetime of 4.9 × 10^−7^ s^45–48^. Such macroscopic bit-flip lifetimes pave the way to build logical qubits with applications-relevant logical error rates by correcting only phase-flip errors, thereby reducing the overhead of quantum error correction. The simplest way to achieve this is to concatenate cat qubits in a repetition code protecting against phase-flip errors, an architecture that has been the focus of many recent theoretical proposals and optimizations^41,49–53^ (Supplementary Discussion IA).

In this paper, we propose an LDPC architecture of cat qubits. This combines the improved encoding rate of LDPC codes and the hardware-efficient intrinsic protection of cat qubits. This leads to a fault-tolerant quantum computing architecture with extremely low qubit overhead, and a hardware complexity similar to the surface code architecture. The central idea we exploit is that it is possible to construct 2D codes outperforming the 1D repetition code without sacrificing locality. Indeed, the “classical version” of the BPT bound^23^ is $k\sqrt{d}=O(n)$ for 2D codes, which repetition codes (*k* = 1, *d* = *n*) are far from saturating. Note that, similar to the assumptions leading to the BPT bound, *2D local* means that the stabilizers of the code act locally on the qubits arranged in a 2D grid (here, at most next-nearest neighbour) which does not necessarily imply that the connectivity graph is planar. While some of the codes we propose do not have planar connectivity graphs, the locality property still makes it relatively easy to implement using a flip-chip technology (as discussed in Supplementary Discussion IV). Our numerical search for 2D local LDPC codes with favorable parameters allows us to identify codes with the desired distances  ≈ 10 − 30 that exhibit up to 5.5 × higher encoding rates than the repetition code, at the cost of increasing the weight of stabilizers from two to four. Furthermore, we show how the lattice surgery schemes of the repetition code architecture^51^ can be adapted to these codes to perform a universal set of fault-tolerant logical gates without sacrificing locality. We provide in Table 1 a comparison of the footprint and the key technological assumptions of our architecture alongside some of the best alternatives to build a 100-logical qubit quantum processor with a logical error rate *ϵ*_*L*_ ≤ 10^−8^ using superconducting circuits. These numbers correspond to the entrance into the “fault-tolerant” regime where quantum hardware becomes able to solve useful computational problems that are beyond the reach of classical methods, such as open problems in quantum materials^54^, the simulation of the dynamics of a 2D Hubbard model with 10 × 10 lattice sites^7^ or the simulation of 3D spinful jellium^55^.

**Table 1:** Technological cost versus footprint reduction trade-offs

	Surface code + sc qubits^65,78^	High-rate qLDPC codes + sc qubits^20^	Repetition code + cat qubits^51^	High-rate LDPC code + cat qubits (this work)
Short-range interactions	yes (2D)	no (2D)	yes (1D)	yes (2D)
Qubit connectivity degree	3–4	6	2	4
*N*_*L*_ = 100 footprints				
$$\left.\begin{array}{l}\epsilon=1{0}^{-3}\hfill\\ {\kappa }_{1}/{\kappa }_{2}=1{0}^{-4}\end{array}\right\}\to {\epsilon }_{L}\le 1{0}^{-8}$$	*N* = 33,700 –	2400 (N/14) –	– 2100 (N/16)	– 758 (N/44)

The recently proposed architectures are compared to the surface code + superconducting (sc) qubits. The technological cost includes the locality of qubit interactions and the qubit connectivity degree on the chip (generally corresponding to the weight of the stabilizers). The footprint corresponds to the total number of physical qubits *N* required to implement a processor with *N*_*L*_ = 100 logical qubits, with a logical error rate per code cycle and per logical qubit of *ϵ*_*L*_ ≤ 10^−8^, assuming circuit-level noise with a physical error rate *ϵ* = 10^−3^ for generic superconducting (sc) qubits or a ratio *κ*_1_/*κ*_2_ = 10^−4^ for cat qubits, where 1/*κ*_1_ is the single-photon lifetime of the resonator hosting the cat qubit and *κ*_2_ the two-photon stabilisation rate of the cat qubit. This ratio determines the error models of all cat qubit operations^41,50^. Concretely, the value *κ*_1_/*κ*_2_ = 10^−4^ corresponds to a circuit-level error model with state preparation and measurement infidelities *ϵ*_SPAM_ = 1.1 × 10^−3^, CNOT gate infidelity of *ϵ*_CNOT_ = 1.6 × 10^−2^ and idling errors of *ϵ*_idling_ = 1.1 × 10^−3^, that is, all operations are noisier than for a depolarizing error model with strength *ϵ* = 10^−3^. The reported footprint of our architecture corresponds to the case where bit-flip errors are entirely suppressed using the passive error correction provided by cat qubits, such that the active error correction code is devoted exclusively to phase-flip error correction. The *ϵ*_*L*_≤10^−8^ target corresponds to a physical bit-flip time at the level of the cat qubit of *T*_*X*_ ~ 13 minutes, which we deem to be reasonably close to the bit-flip times already demonstrated experimentally^46–48^ (Supplementary Discussion IB).

## Results

### High-rate local phase-flip codes

We turn our attention to the search for small phase-flip LDPC codes with higher encoding rates than repetition codes for relevant code distances. As cat qubits are protected against bit-flip errors, only *Z*-type errors need to be corrected. Thus we look for codes for which the stabilizer group is a subgroup of the Pauli group with *X*-type stabilizers exclusively and where codestates can be naturally expressed in the $\left\vert \pm \right\rangle$ basis.

**Fig. 1:**
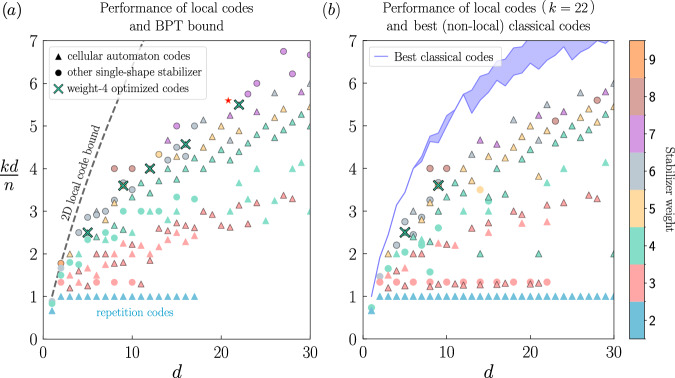
Overhead reduction factor over the repetition code *kd*/*n* of 2D local phase-flip codes, as a function of the code distance *d.* **a** Each marker represents a single code, and the color indicates the stabilizer weight. The circular and triangular markers indicate codes with local stabilizers fitting within a 3 × 3 grid of data qubits, which are invariant under vertical and horizontal translations on the grid of qubits. By construction, the stabilizers have weight at most 9, and at most next-nearest neighbor physical extension. We systematically test all possible stabilizer shapes among the 512 possibilities {*I*, *X*}^⊗9^ but only the best codes are displayed. The triangles highlight the family of cellular automaton codes, which feature attractive properties for the quantum architecture (see main text). All codes are constructed on lattices of size *H* × *L* ≤ 17 × 17 with *n* = *HL* data qubits, with periodic boundary conditions on the lateral sides which allows us to avoid specifying the precise shape of the code lateral boundaries. However, this condition can be removed to retrieve local codes (Supplementary Discussion IIA). The codes identified by a cross have been found by allowing the shape of the (weight-4) stabilizers to differ at each row of the lattice. The 2D code performance upper bound is also indicated^23^. Markers with an outline indicate stabilizer shapes spanning three rows which tend to exhibit superior performance for almost all distances. **b** Performance of 2D local phase-flip codes encoding *k* = 22 logical qubits as a function of the code distance. The codes are constructed on lattices of size up to *L* = *H* = 34, with periodic boundary conditions on the lateral sides. To compare the performance of our local codes in an absolute sense, we represent, for a fixed number of logical qubits, the theoretical upper bound for the overhead reduction factor together with the best-known classical codes^79^ (thick blue curve, where the top part indicates the best possible code and the bottom part the best-known code). Quite remarkably, for the parameters *k* and *d* considered, our local codes are close to the best (potentially non-local) existing classical codes.

We impose the 2D local constraint and consider stabilizers limited by next-nearest neighbor interactions only. This is in contrast with previous approaches^20,25^ that required long-range interactions. We first consider codes where all the stabilizers are generated by horizontal and vertical translations of a single generator stabilizer on the 2D lattice, so that the “shape” of the stabilizer is unique (for instance,  is the repetition code X stabilizer shape and  corresponds to the X stabilizer shape of the surface code). The results are depicted in Fig. 1(a), where the best codes are shown. We evaluate the merit of the different codes using the value of *kd*/*n*, quantifying the overhead improvement over the repetition code, for which *kd*/*n* = 1. Therefore, the value of *kd*/*n* for other phase-flip codes can be interpreted as the overhead reduction factor of the number of physical qubits to implement a given number of logical qubits with a specific distance.

**Fig. 2:**
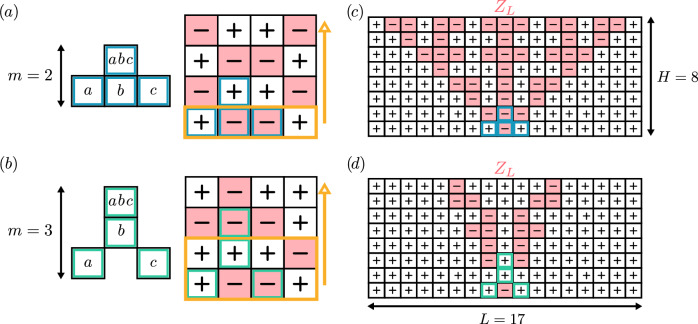
Example of phase-flip cellular automaton codes on a *H* × *L* lattice of data qubits. Cellular automaton codes are a family of local codes characterized by “pointed''-shape stabilizers, acting on a single qubit of the top row of their support. Thus, for a stabilizer shape spanning *m* rows, the 2^*k*^ codewords are uniquely determined by the $\left\vert \pm \right\rangle$ states of the bottom (*m*−1) rows of qubits and therefore correspond to a code of dimension *k* = (*m* − 1)*L*. Indeed, an entire codeword can be constructed row by row by successive applications of the cellular automaton rule corresponding to the stabilizer, as depicted (yellow arrow) for (**a**) a (*m* = 2, *L* = 4) code encoding 4 logical qubits and (**b**) a (*m* = 3, *L* = 4) code encoding 8 logical qubits. Consequently, the codewords can be interpreted as the vertical evolution of a 1D cellular automaton, with time progressing upwards and the stabilizers dictating the evolution rule. In (c-d), we represent a *Z*_*L*_ logical operator of minimal weight for the two codes corresponding to the stabilizer shapes in **a, b**, respectively. The corresponding *X*_*L*_ logical operator corresponds to a single *X* on the physical qubit at the bottom row of the lattice in the *Z*_*L*_ support. We numerically observe that, for a lattice with sufficient width (about twice its height) or when periodic boundary conditions are removed (Supplementary Discussion IIA), the codewords with the smallest Hamming weight, which determine the distance, are the ones with a single $\left\vert -\right\rangle$ state in the bottom row for most cellular automaton codes. Note that any horizontal shift of this logical operator remains a valid logical operator, thus defining a basis of minimum-weight logical operators. The (*m* − 1)*L* logical qubit *Z*_*L*_ supports of this basis consist of a single $\left\vert -\right\rangle$ state in the *m* − 1 bottom rows and extend in a “fractal” manner to the rows above, justifying the occasional reference to cellular automaton codes as fractal codes^59^. Note that the code (**d**) has a smaller distance than the code (**c**) but this is compensated by a doubled encoding rate, such that the overall overhead reduction factor is larger for the same distance.

Unsurprisingly, codes with high stabilizer weights generally exhibit superior performance. Interestingly, many of the best codes we identified belong to the family of cellular automaton codes^23,56–60^ which are known to have a better asymptotic scaling than repetition codes^23,59^. These codes, identified by triangles in Fig. 1, are characterized by a “pointed” stabilizer shape, *i.e*. the stabilizer non-trivially acts on only one of the qubits in the top row of its support as illustrated in Fig. 2a, b. This property implies a systematic construction of the logical codewords using a cellular automaton rule corresponding to the stabilizer’s shape. The number of encoded logical qubits *k* can then be arbitrarily tuned by varying the width of the lattice.

Cellular automaton codes have been known to achieve a good performance, due to the “fractal” nature of the support of logical operators, which yields large distances compactly^23,59,60^ as shown in Fig. 2c, d. A minimal-weight logical basis of the code can be formed by horizontally translating the logical operator. Increasing the height of the lattice increases the support of the logical operators, and thus the distance, maintaining a constant number of logical qubits as long as the width remains unchanged. Remarkably, for a small number of logical qubits and relevant distances, the codes we found are close to the best (potentially non-local) existing codes, see Fig. 1b.

We introduce two improvements over previously existing cellular automaton codes. First, by selecting a stabilizer shape extending over *m* = 3 rows instead of *m* = 2, the number of logical qubits is equal to twice the width of the lattice, doubling the rate of the code. For these codes, the distance increases more slowly with the height, as can be seen in Fig. 2d where the support of the logical operators *Z*_*L*_ is smaller. However, as more logical qubits are encoded, the overall code parameters are improved as identified in Fig. 1 by markers with an outline for codes where *m* = 3. Second, we extended the search to cellular automaton codes for which the stabilizer shape in each row of the lattice is allowed to differ. We focused on weight-4 stabilizers, which we deem to be the best compromise between stabilizer weight and the overhead reduction factor *kd*/*n* based on the search results. We identified the optimal stabilizer shapes maximizing the cellular automaton codes performance. To assess the gain in performance due to these optimizations, these new codes are shown (marked by crosses) in Fig. 1. The optimization significantly improves the performance: the codes with weight-4 stabilizers of varying shapes achieve comparable performances as codes with a single stabilizer shape of weights 5 to 7. Their specific structure can be found in Table 2. In particular, the [136, 34, 22] code reduces the overhead by a factor *kd*/*n* = 5.5 for distance *d* = 22, nearly doubling the performance over the previously existing cellular automaton codes with only 4 physical qubits per logical qubits.

**Table 2:** Optimized cellular automaton codes compared to the repetition code (first line)

$[n,k,d],\ell \in {\mathbb{N}}$	*kd*/*n*	(*H*, *L* = L^*^ + *ℓ*)	Stabilizer shapes (bottom to top)
[*ℓd*, *ℓ*, *d*]	1	(*H* = *d*, *ℓ*)	
[20 + 4*ℓ*, 10 + 2*ℓ*, 5]	2.5	(4, 5 + *ℓ*)	
[55 + 5*ℓ*, 22 + 2*ℓ*, 9]	3.6	(5, 11 + *ℓ*)	
[78 + 6*ℓ*, 26 + 2*ℓ*, 12]	4	(6, 13 + *ℓ*)	
[119 + 7*ℓ*, 34 + 2*ℓ*, 16]	4.6	(7, 17 + *ℓ*)	
[136 + 8*ℓ*, 34 + 2*ℓ*, 22]^⋆^	5.5	(8, 17 + *ℓ*)	

The codes are implemented on a *H* × *L* lattice of data qubits with periodic boundary conditions on the lateral sides. These codes feature a different stabilizer shape for each row, except for the repetition code in the first row, where each of the *l* columns is an independent distance *d* repetition code. L^*^ corresponds to the minimal width *L* required to achieve the full code distance set by the value of *H*: increasing values of *L* ≥ L^*^ (*H* fixed) yields codes with the same distance *d*, but with code dimension *k* = (*m* − 1)*L* and code size *n* = *HL*, therefore, identical overhead reduction ratio *kd*/*n*. Here and throughout the paper, the (red) star identifies the [136 + 8*ℓ*, 34 + 2*ℓ*, 22] code family. For the sake of completeness, we mention that in rare cases, interference phenomena reduce the distance for very specific values of *L* > L^*^. These features disappear when the periodic boundary conditions are removed (Supplementary Discussion IIA).

### Numerical estimation of the logical error probability

We now quantify more precisely the logical error probability of the optimized codes which exhibit the most favorable overhead reduction factor *kd*/*n*. The total logical error probability is given by ${\epsilon }_{L}\approx {p}_{{X}_{L}}+{p}_{{Z}_{L}}$ (as ${p}_{{Y}_{L}}={p}_{{X}_{L}}{p}_{{Z}_{L}}\ll {p}_{{X}_{L}},{p}_{{Z}_{L}}$), where ${p}_{{X}_{L}}$ and ${p}_{{Z}_{L}}$ are the probabilities of logical bit-flip and phase-flip errors per code cycle. To compare the different codes, the logical errors ${p}_{{X}_{L}}$ and ${p}_{{Z}_{L}}$ are normalized by the number of logical qubits encoded. Since phase-flip codes do not protect against bit-flip errors, we estimate the logical phase-flip and bit-flip probability separately. ${p}_{{X}_{L}}$ is simply given by *N*_cat qubits_ × *p*_*X*_/*k*, where *N*_cat qubits_ is the total number of cat qubits and *p*_*X*_ is the physical bit-flip error probability per error correction cycle. We evaluate ${p}_{{Z}_{L}}$ with a Monte Carlo method and derive a phase-flip threshold. Note that overall this architecture does not have a conventional threshold, as the physical phase-flip rate of cat qubits increases linearly with the average photon number $\bar{n}$, eventually surpassing the phase-flip threshold of the code. But in practice it enables us to reach sufficiently low logical error rates with a competitive overhead compared to other approaches (Supplementary Discussion IA).

We compare the optimized codes of Table 2 to the repetition code family under a *cat qubit circuit-level* error model, which includes errors at all locations in the circuit. The precise error models are obtained using master equation simulations of noisy operations on cat qubits^41,50^ parameterized by the relevant ratio *κ*_1_/*κ*_2_, where *κ*_1_ is the single-photon loss rate of cat qubits and *κ*_2_ is the two-photon stabilization rate (Supplementary Discussion IB).

**Fig. 3:**
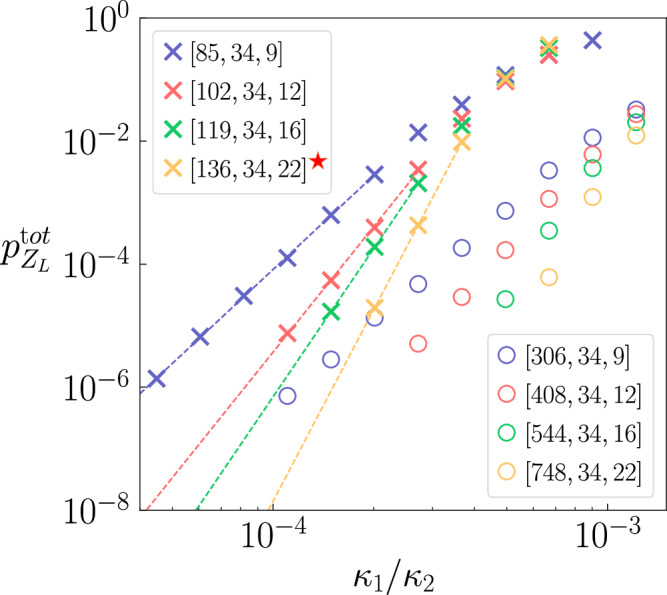
Logical phase-flip error probability of high-rate phase-flip codes compared to the repetition code. The probability is normalized by the number of logical qubits, calculated after *d* error correction cycles (${p}_{{Z}_{L}}^{{{{\rm{tot}}}}}$) and plotted as a function of the physical error probability, under a cat qubit circuit-level error model. Repetition codes are identified by circles and optimized codes from Table 2 by crosses. The *d* rounds are decoded using the belief propagation + ordered statistics decoding (BP+OSD) decoder^73,74^. The decoder succeeds if the proposed correction successfully removes the errors, otherwise it introduces a logical error. The circuit is sampled until 100 logical errors are observed, and the probability of a logical phase-flip error after *d* rounds is estimated as ${p}_{{Z}_{L}}^{{{{\rm{tot}}}}}=1-{(1-100/N)}^{1/k}$ where *N* is the total number of samples (the standard deviations, not depicted in the figure, are approximately equal to ${p}_{{Z}_{L}}^{{{{\rm{tot}}}}}/10$, smaller than the marker size). The logical phase-flip error per round is then estimated as ${p}_{{Z}_{L}}\approx {p}_{{Z}_{L}}^{{{{\rm{tot}}}}}/d$. We observe that codes with identical distances have a similar scaling but the phase-flip threshold is lower for the cellular automaton codes. We attribute this effect to the depth of the weight-4 stabilizer measurement circuits compared to the weight-2 stabilizers of the repetition codes. The fit is used to extrapolate the logical error rate of the *d* = 22 code to *κ*_1_/*κ*_2_ = 10^−4^ (Supplementary Discussion IIB).

We plot the logical phase-flip error probability as a function of physical error probability in Fig. 3. The phase-flip threshold of cellular automaton codes is approximately 3 times lower, fitting to the ansatz ${p}_{{Z}_{L}}=0.1{(1613{\kappa }_{1}/{\kappa }_{2})}^{0.94\lfloor \frac{d+1}{2}\rfloor }$ compared to $0.07{(486{\kappa }_{1}/{\kappa }_{2})}^{0.94\lfloor \frac{d+1}{2}\rfloor }$ for the repetition code. We attribute this to the fact that the syndrome measurement circuit is deeper for these codes, as the weight-four stabilizers require four CNOT gates to be measured instead of two for the weight-two repetition code (Supplementary Discussion IIB). However, for a targeted logical error rate *ϵ*_*L*_ = 10^−8^, cellular automaton codes still provide a significant overhead advantage over repetition codes. Indeed, at *κ*_1_/*κ*_2_ = 10^−4^, we estimate the logical phase-flip error probability per cycle and normalized by the number of logical qubits at ${p}_{{Z}_{L}}=6.4\times 1{0}^{-10}$ with the [136, 34, 22] code. The simulations were performed with a number of photons $\bar{n}=11$ so that the logical bit-flip probability is given by ${p}_{{X}_{L}}=1.8\times 1{0}^{-9}$.

As the number of logical qubits can be arbitrarily tuned with cellular automaton codes, the [165 + 8*ℓ*, 34 + 2*ℓ*, 22] code family, the extended version of the [136, 34, 22] code without periodic boundary conditions (Supplementary Discussion IIA), can be used to encode 100 logical qubits in a [429, 100, 22] code, for a total number of 758 cat qubits including ancilla qubits compared to 2100 for the repetition code. The code is expected to achieve a total error probability *ϵ*_*L*_ = 2.5 × 10^−9^. Note that a lower value of *κ*_1_/*κ*_2_ would increase the overhead advantage on the repetition code. However, the number of physical data cat qubits per logical qubit of this code is 4.3 (for a code of distance 22), such that this code is already extremely efficient.

### Fault-tolerant universal logical gate implementation

"Block” codes, where the coding blocks contain several logical qubits, allow for a compact encoding, but the price to pay is that it is more difficult to manipulate logical information. Indeed, unlike the case where only one logical qubit is encoded per block, here the supports of the logical operators corresponding to different logical qubits overlap, such that each physical qubit belong to the logical operator support of distinct logical qubits. Therefore, it is not straightforward to individually address logical qubits.

The initial proposals for “block” codes were based on teleportation^61,62^. In order to reduce the overhead of these protocols, more recent works propose to use state preparation and teleportation^26,63^ or, more efficiently, to adapt the lattice surgery techniques^29,33^ developed for the surface code^64^. Alternatively, code deformation methods can be used^27,28^.

**Fig. 4:**
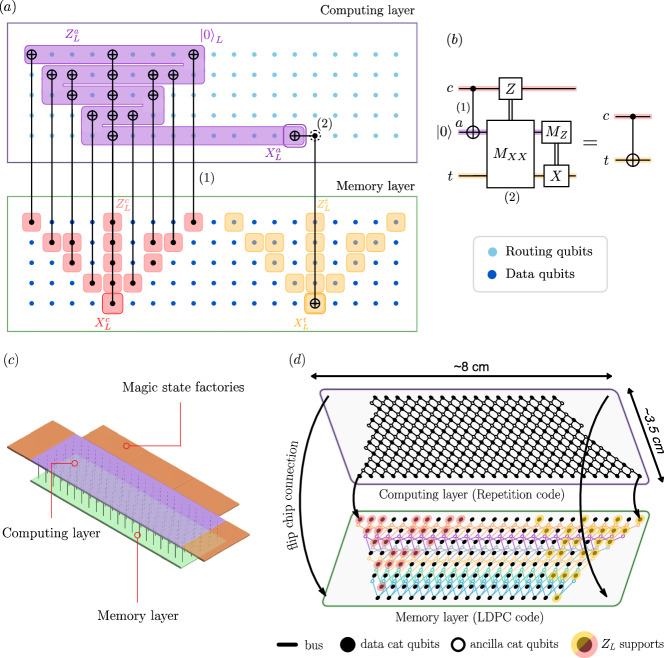
Logical CNOT gate between two logical qubits encoded in the cellular automaton code. Figure **b** presents the lattice surgery circuit used^64^. As depicted in Figure **a**, the implementation requires routing qubits encoded in the repetition code. First, a logical ${\left\vert 0\right\rangle }_{L}$ repetition code is prepared. A logical CNOT gate is performed between the control logical qubit and the logical ${\left\vert 0\right\rangle }_{L}$. This is followed by an *M*_*XX*_ measurement between the logical ${\left\vert 0\right\rangle }_{L}$ and the target logical qubit. This process includes preparing a routing ancilla (dashed circle) in the $\left\vert+\right\rangle$ state, performing two CNOTs with the logical ${\left\vert 0\right\rangle }_{L}$ and the logical ${X}_{L}^{t}$ of the target logical qubit, and finally measuring the ancilla. The result is the *M*_*XX*_ measurement and the procedure is repeated *d* times to ensure fault-tolerance. Finally, the logical ancilla is measured in the *Z* basis and some Pauli corrections are applied depending on the *M*_*XX*_ and *M*_*Z*_ measurements. **c** Layout overview of the quantum processor with a bilayer architecture required for performing operations on 2D local phase-flip codes. CNOT gates are allowed between corresponding vertical qubits in the memory layer and the computing layer. Magic state factories are located on the side of the computing layer, and the states can be used to perform the corresponding gates in the memory layer through Clifford circuits via the computing layer. **d** Layout of a [165, 34, 22]^⋆^ cellular automaton code corresponding to the optimized code of Table 2 for *H* = 8 but without periodic boundary conditions. Flip chip technology enables connections between corresponding qubits in the memory layer and the computing layer. The weight-4 stabilizers of the code feature crossing cables but this can be circumvented by passing through the other plane. The trapezoidal shape enables to remove the periodic boundary conditions and ensures that the support of the *Z*_*L*_ logical operators on the side (in red and yellow) achieve full distance in order to preserve the code parameters (Supplementary Discussion IIA). All data qubits and ancilla qubits are represented (except for the magic state factories) for a total of 296 qubits for the memory layer and 311 for the computing layer.

Here, we propose to implement a universal set of logical gates on our phase-flip LDPC codes with a two-layer architecture, which can be realized with existing flip-chip technologies (Supplementary Discussion IV). The lower layer is a “memory” layer that contains the logical qubits encoded in high-rate local phase-flip codes, and the upper layer is a “computing” layer that contains logical routing qubits and magic state factories encoded in repetition codes, as depicted in Fig. 4c. Each of the data qubits of the memory layer is connected to the corresponding data qubit of the computing layer, in order to implement physical CNOT gates. This construction only increases the connectivity of the logical data qubit of the memory layer by one while retaining locality. The ancillary logical qubits encoded in repetition codes are used to implement a universal set of logical gates on the qubits of the memory layer by adapting the schemes proposed for the repetition code architecture. We redirect the reader to the refs. ^41,50,51^ for a detailed description of the logical operations and here focus only on the differences due to the block code encoding. More precisely, the set of logical states $\{{\left\vert+i\right\rangle }_{L},{\left\vert {\mbox{C}}Z\right\rangle }_{L},{\left\vert {{\mbox{CC}}}X\right\rangle }_{L}\}$ can be prepared using fault-tolerant logical measurements on repetition codes^50^. This set of states allows for a universal gate set when supplemented with the set of operations $\{{{{{\mathcal{P}}}}}_{{\left\vert 0\right\rangle }_{L}},{{{{\mathcal{P}}}}}_{{\left\vert+\right\rangle }_{L}},{Z}_{L},{X}_{L},\,{{\mbox{C}}}\,{X}_{L},{{{{\mathcal{M}}}}}_{{Z}_{L}},{{{{\mathcal{M}}}}}_{{X}_{L}}\}$, some of which can be directly realized in the memory layer, as we now detail. We focus on cellular automaton codes with the stabilizer shape  as in Fig. 2a for simplicity but the schemes are general and can be realized for all of the codes of Fig. 1.

Pauli preparations and measurements can be performed on every logical qubit by leveraging stabilizer measurements^65^ or by utilizing ancilla qubits (Supplementary Discussion IIIA). A logical CNOT gate between two logical qubits of the memory layer can be directly implemented using the computing layer, as shown in Fig. 4a, with the lattice surgery scheme described in^64^, depicted in Fig. 4b. Note that this scheme can be extended to realize multiple-target controlled NOT gates $\,{{\mbox{C}}}\,{X}_{L}^{k}$^51^, or several CNOT gates sharing the same target in parallel. However, two CNOTs with overlapping control qubits and different target qubits cannot be realized simultaneously as the routing qubits used for one CNOT are unavailable for the other. As a consequence, a high level of parallelization is possible, but with some minor constraints. Finally, logical magic states are prepared in dedicated magic state factories in the computing layer as depicted in Fig. 4c and teleported to perform the operations required to complete the universal set of gates with minor adaptations of the techniques used for the intra-block C*X*_*L*_ gate (Supplementary Discussion IIIB).

## Discussion

The use of extremely biased-noise qubits concatenated in high-rate phase-flip LDPC codes opens a new path towards hardware-efficient quantum computing. The proposed architecture is local in 2D with low-weight stabilizers, constraints similar to the surface code. This contrasts with standard non-biased qubits which necessarily require technologically challenging long-range connectivity to exploit high-encoding rate quantum LDPC codes. A universal and fault-tolerant set of logical gates can be implemented using local physical operations by adapting lattice surgery schemes. An important assumption is that the bit-flip error rate is suppressed at the desired level by the cat qubits alone. This has already been demonstrated during idling and Z gates^46–48^, and recent promising experimental results demonstrated a bias-preserving CNOT gate^66^. Rapid progress in cat qubit engineering has allowed to achieve a ratio *κ*_1_/*κ*_2_ = 6.5 × 10^−3 ^^48^ and we deem a value around *κ*_1_/*κ*_2_ = 10^−4^ may be achieved when the platform reaches its experimental maturity (see Supplementary Discussion IB for details about the hardware hypothesis). Furthermore, this assumption could be relaxed due to recent theoretical progress (Supplementary Discussion IC). The two layers of the architecture can be realized with a flip-chip technology^67,68^ (Supplementary Discussion IV) as shown in Fig. 4d. In order to quantify how the gains in memory reduce resources requirements for fault-tolerant quantum algorithms, we benchmark our architecture against the repetition cat code architecture which can be used to factorize RSA-2048 integers in 4 days using 350000 cat qubits^51^. We estimate that, under the same hardware assumptions (*κ*_1_/*κ*_2_ = 10^−5^), the improvements proposed in this paper would reduce this number to less than 100000 cat qubits, and 7 days of computation.

## Methods

### Architectures comparison

Comparing architectures that do not rely on the same types of qubit is a somewhat difficult task because one cannot directly use the same error models to make an apples-to-apples comparison. However, it remains an interesting exercise that sheds light on the importance of the assumptions made for each architecture, and allows for a better understanding of the significance of experimental progress for each physical platform. We first detail how the figures in Table 1 were obtained, before discussing several recent theoretical proposals to further optimise the performance of the cat qubit architecture.

### Surface code

Assuming a circuit-level depolarizing noise where all of the operations in the syndrome measurements circuits are noisy, with identical infidelity *ϵ*, and using a minimum-weight perfect matching decoder, the logical error probability per code cycle *ϵ*_*L*_ is well approximated by^69,70^

1 $${\epsilon }_{L}=0.1{(100\epsilon )}^{\lfloor \frac{d+1}{2}\rfloor }.$$

For *ϵ* = 10^−3^, a logical error rate *ϵ*_*L*_ = 10^−8^ is achieved for a code distance *d* = 13. With these numbers, one can encode *N*_*L*_ = 100 logical qubits with *N* = *N*_*L*_(2*d*^2^ − 1) = 33,700 physical qubits (including ancilla qubits).

### Small qLDPC code

We focus for this example on the [[144, 12, 12]] qLDPC code introduced in ref. ^20^. This code is a quasi-cyclic code of CSS type with stabilizer operators of weight 6. The Tanner graph of this code has thickness two, which implies from a technological point of view that the code can be implemented using two planar layers of couplers that do not intersect (see ref. ^20^ for a detailed discussion). Under a circuit-level depolarizing noise of strength *ϵ* and using a BP+OSD decoder, the logical error probability per code cycle and normalized by the number of logical qubits *ϵ*_*L*_ is well approximated by^20^

2 $$12{\epsilon }_{L}={\epsilon }^{5}{e}^{{c}_{0}+{c}_{1}\epsilon+{c}_{2}{\epsilon }^{2}},$$

where *c*_0_ = 18.04, *c*_1_ = 1337, *c*_2_ = − 96007. Thus, *ϵ* = 10^−3^ corresponds to a logical error rate *ϵ*_*L*_ = 2 × 10^−8^ and the total number of physical qubits required to implement *N*_*L*_ = 100 logical qubits is *N* = 2*nN*_*L*_/12 = 2400.

### Cat qubits and repetition codes

Assuming all of the operations of the repetition code (ancilla cat state preparation, CNOT gates, and ancilla measurement, see^41,44^ for a detailed review) are realized in time *T* = 1/*κ*_2_, where *κ*_2_ is the two-photon dissipation rate, and assuming that the only error channel is single-photon loss at a rate *κ*_1_, the logical error probability per code cycle *ϵ*_*L*_ is well-approximated by^51^

3 $${\epsilon }_{L}=5.6\times 1{0}^{-2}{\left(\frac{{\bar{n}}^{0.86}{\kappa }_{1}/{\kappa }_{2}}{1.3\times 1{0}^{-2}}\right)}^{\frac{d+1}{2}}+2(d-1)\times 0.50{e}^{-2\bar{n}},$$

where *d* is the repetition code distance (against phase-flip) and $\bar{n}$ is the mean photon number of the cat, playing the role of the “distance” against bit-flips. Note that here ${\epsilon }_{L}={\epsilon }_{L}^{Z}+{\epsilon }_{L}^{X}$ corresponds to the total logical error rate, while in the two previous examples *ϵ*_*L*_ is the logical error probability of either logical bit-flips or phase-flips (identical). Assuming a ratio *κ*_1_/*κ*_2_ = 10^−4^, one achieves a logical error rate *ϵ*_*L*_ = 2.8 × 10^−9^ + 2.7 × 10^−9^ = 5.5 × 10^−9^ using code distances $(\bar{n},d)=(11,11)$, which corresponds to a total number of cat qubits *N* = (2*d* − 1)*N*_*L*_ = 2, 100. Note that these assumptions translate (see Supplementary Table 1) into state preparation and measurement infidelities of *ϵ*_SPAM_ = 1.1 × 10^−3^, CNOT gate infidelity of *ϵ*_CNOT_ = 1.6 × 10^−2^ and idling errors of *ϵ*_idling_ = 1.1 × 10^−3^, that is, all operations are noisier than for a depolarizing error model with strength *ϵ* = 10^−3^.

### Efficient computation of the code distance

In the exhaustive numerical search for the best phase-flip local LDPC codes, we considered all 511 possible stabilizer shapes fitting withing a 3 × 3 square of data qubits. We also varied the dimension of the 2D grid up to *H* × *L* ≤ 17 × 17. Each possible stabilizer shape yields a code for which we calculate the dimension *k* and distance *d*. Since these codes are linear, one can put their parity-check matrix (defined by the stabilizers) into normal form to obtain a basis of *k* codewords of the codespace. The distance of the code may then be computed exactly by constructing the 2^*k*^ codewords, as the code distance corresponds to the minimal non-zero Hamming weight of the codewords.

Computing the exact distance of a classical code is known to be an NP-hard problem^71^. In this work, we relied on two approaches: the first one simply consists in enumerating all of the 2^*k*^ − 1 nonzero codewords and computing the minimum Hamming weight. This method becomes very costly for *k* ≥ 34, even if cellular automaton codes have the nice advantage that the systematic construction of the codewords increases the computation speed.

The second method is to use a SAT solver. SAT solvers have been optimized to solve the boolean satisfiability problem, *i.e*. finding possible values for boolean variables to satisfy a set of constraints. We define these variables and constraints with the generator matrix *G* of the code, which gives a basis of *k* codewords, where any codeword *c* can be written as $c=\mathop{\sum }_{i=1}^{k}{b}_{i}{G}_{i}$. *G*_*i*_ is the codeword corresponding the i-th row of the matrix *G* and *b*_*i*_ are the boolean variables of our problem.

Given that the goal is to find the minimal Hamming weight of a codeword, we impose the following constraints for a given tested distance ${d}^{{\prime} }$: at least one *b*_*i*_ must be non-zero and the $\mathop{\sum }_{i=1}^{k}{b}_{i}{G}_{i}$ must be at most ${d}^{{\prime} }$. If the SAT solver finds a combination of *b*_*i*_ which is a solution, then the distance of the code $d\le {d}^{{\prime} }$, otherwise $d > {d}^{{\prime} }$. The minimal value ${d}^{{\prime} }$ for which the problem is satisfied is the code distance *d*, found using a dichotomic search over ${d}^{{\prime} }$.

Using this method and the z3 SAT solver^72^, we were able to compute the exact distance of some codes up to *k* = 62. This is possible because the SAT solver does not go through the entire code space but explores the space of possibilities given the constraints in a more subtle way. We numerically observed that the SAT solver is not always faster than the brute force method, however, we did not find any simple criteria to predict which method is most efficient for a given code, as SAT solvers are complex algorithms.

### Efficient code optimisation

In this section, we detail the procedure to find the optimal stabilizer shapes of cellular automaton codes. The search space consists only of codes with “pointed” stabilizer shapes so that any codeword is uniquely determined by its values at the bottom rows of the lattice.

Varying the stabilizer shapes of the code will not change the number of encoded logical qubits for a given width and height but can increase the distance of the code. This can be understood from Fig. 2c-d, showing a codeword of minimal Hamming weight, which consists of a single $\left\vert -\right\rangle$ state in the bottom rows. The Hamming weight, *i.e*. the number of $\left\vert -\right\rangle$ states, in this codeword will never exceed the number of qubits within the triangle area. But by changing the stabilizer shapes (*i.e*. the rules of the cellular automaton), we may increase the number of $\left\vert -\right\rangle$ states.

We further restrict the search space to a unique stabilizer shape in each row of the lattice. This guarantees that the search remains tractable, while we empirically found that, for smaller instances, removing this constraint does not bring a significant advantage. Brute-forcing the problem becomes quickly intractable, so we used a SAT solver for the largest instances^72^.

We first fix the width and height of the lattice. The boolean variables correspond to the allowed stabilizer shapes (with several boolean variables per shape) and 2^*k*^ constraints impose that all codewords have a Hamming weight larger than a tested distance ${d}^{{\prime} }$. If the solver is able to find stabilizer shapes, which verify all the constraints, it corresponds to a code with distance ${d}^{{\prime} }\ge d$. By progressively increasing ${d}^{{\prime} }$, we are able to find the maximum distance attainable for a cellular automaton code of a given height.

The constraints (i.e. the Hamming weight of the codewords) need to be expressed with boolean variables. Thanks to the structure of cellular automaton codes, this task just consists of applying the cellular automaton rules from a given bit-string in the bottom rows conditionally on the boolean variables. This method gives relatively simple expressions compared to a general code.

### BP+OSD decoding

In this section, we briefly present the BP+OSD decoder and the parameters we chose. This decoder was first introduced in^73^ and a more efficient variant of the ordered statistics decoding (OSD) was proposed in^74^. The first step of the decoding process is belief propagation where an error probability is assigned to every qubit knowing the syndrome. This marginal probability is calculated iteratively until a maximum number of steps. If the proposed correction is in agreement with the given syndrome, the decoding process stops there. Otherwise, the OSD routine is called. The qubits for which the value is the most reliable at the end of BP are fixed, and a greedy search is performed on the other qubits for the codeword with the smallest Hamming weight and which is in agreement with the syndrome. The OSD order designates the number of qubits involved in this greedy search and the number of combinations tested increases exponentially with the order.

**Fig. 5:**
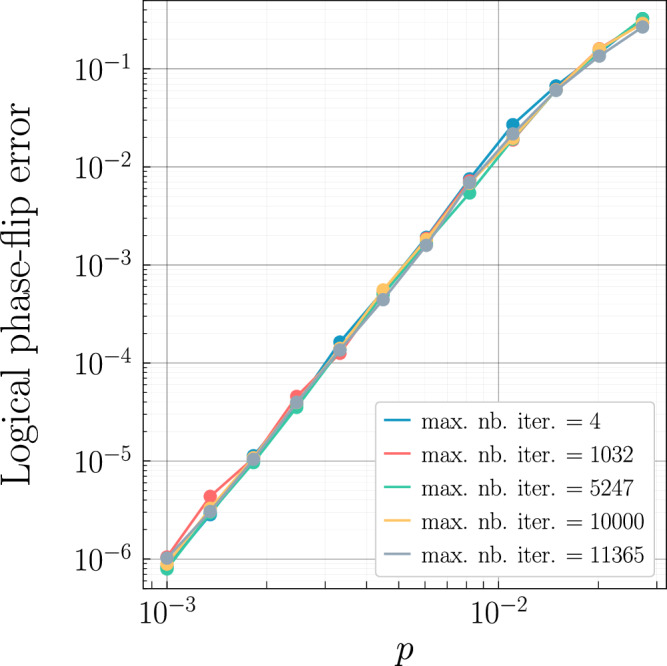
Logical phase-flip error rate for the cellular automaton code with the stabilizer shape presented in Fig. 2a. The lattice size is set as *H* = 3 and *L* = 10 which corresponds to a code of distance 7. The circuit-level noise model is considered and *p* designates the physical error rate on every gate and idle location in the circuit. The maximum number of iterations of the belief propagation varies showing that this parameter does not play a significant role in the decoding process. The min-sum method is used and the scaling factor is set to 0.625.

We chose the “min-sum” variant of belief propagation and we have observed no significant difference between the various methods ("product-sum”,"min-sum”) in the case of the cellular automaton code decoding process. Additionally, the decoding is slightly better when the scaling factor is chosen at 0.625 as proposed in^73^ when the “min-sum” method is used. Finally, the number of iterations does not appear to play a significant role either (even when set to 4, which corresponds to the cycle length in the Tanner graph of the code) as shown in Fig. 5. The numerical results reported in our figures are obtained with a maximum number of iterations of 10,000.

Regarding the OSD routine, increasing the OSD order increases the performance of the decoder but at an exponential cost in time. We confirm, as that was stated in^74^, that the “combination sweep” method surpasses the “exhaustive method” for an equivalent number of configurations tested. We set the OSD order to 60, which gives a number of configurations to search through of 1770. The question of real-time decoding and a possibly faster or more efficient decoder for these very structured codes remains an open question.

### Factoring 2048-bit RSA integers

In order to estimate the total number of cat qubits required to factorize 2048-bit RSA integers, we rely on the implementation of Shor’s algorithm described in^75^ and adapted to the cat qubit architectures in^51^, as the set of logical gates is identical. In this previous work, logical gates are not parallelized, in order to minimize as much as possible the space overhead (number of logical qubits) at the cost of increasing the time overhead (the runtime of the algorithm). We follow the same implementation, such that the fact that some logical CNOT gates might not be executed in parallel on our codes is not an issue here.

In order to provide a fair comparison of the reduction in overhead, we use the exact same hardware assumptions and error models as in^51^. We therefore assume a ratio *κ*_1_/*κ*_2_ = 10^−5^, and an average photon number $\bar{n}=21$, in order to reach the extremely low logical error probability *ϵ*_*L*_ ≈ 10^−17^ per logical qubit and per code cycle required to run Shor’s algorithm.

Replacing the distance *d* = 15 repetition codes by the dense [24885, 6214, 22] code of the [165 + 8*ℓ*, 34 + 2*ℓ*, 22] family in the memory block, the total number of cat qubits in the architecture decreases from 349133 cat qubits^51^ to 95941 cat qubits (code available here^76^). The computation time increases from 4 to 7 days, as a consequence of the weight-4 stabilizers (instead of weight-2) that increases the error correction cycle time from *T*_cycle_ = 500 ns to *T*_cycle_ = 900 ns.

## Supplementary information

Supplementary Information

Transparent Peer Review file

## Data Availability

The data used in this study are available here^77^.

## Code availability

The code used for numerical simulations, analysis and visualization is available here^77^. The code for Shor’s algorithm for the factorization of 2048 RSA integers resource estimation is available here^76^.
