# Supplementary material for: LDPC-cat codes for low-overhead quantum computing in 2D
Source: Nat Commun. 2025 Jan 26;16:1040. doi: 10.1038/s41467-025-56298-8 (PMC11762751; doi:10.1038/s41467-025-56298-8)
Supplement: Supplementary file 1 — Supplementary Information [file 41467_2025_56298_MOESM1_ESM.pdf]

Supplementary Information for *LDPC-cat codes for low-overhead quantum computing in 2D*

Diego Ruiz,[1,2,*] Jérémie Guillaud,[1] Anthony Leverrier,[3] Mazyar Mirrahimi,[2] and Christophe Vuillot[4]

[1] *Alice & Bob, 49 Bd du Général Martial Valin, 75015 Paris, France*
[2] *Laboratoire de Physique de l'École Normale Supérieure, École Normale Supérieure, Centre Automatique et Systèmes, Mines Paris, Université PSL, CNRS, Inria, 75005 Paris*
[3] *Inria Paris, 48 rue Barrault, 75013 Paris, France*
[4] *Université de Lorraine, CNRS, Inria, LORIA, F-54000 Nancy, France*

(Dated: January 14, 2025)

CONTENTS

I. Cat qubits 1
   A. Repetition code for practical fault-tolerance 1
   B. Current state of the experimental art and further research directions 2
   C. Theoretical optimization 3

II. Cellular automaton codes 3
   A. Removing periodic boundary conditions 3
   B. Logical error simulation details and threshold analysis 4

III. Fault-tolerant operations 4
   A. Pauli measurements and preparations 4
   B. Universal gate set with magic states 6

IV. Chip layout 6

References 7

I. CAT QUBITS

A. Repetition code for practical fault-tolerance

The concatenation of cat qubits with a repetition code protecting against phase-flip errors has been proposed as a hardware efficient approach to fault-tolerant quantum computing [1–6]. The stabilizers of the (distance-d) phase-flip repetition code are $\{X_iX_{i+1}\}_{i\in\{0,\dots,d-2\}}$ and the logical Pauli operators are $X_L = X_0$, $Z_L = \bigotimes_{i=0}^{d-1} Z_i$ and $Y_L = -iZ_LX_L$. The operator X_L has weight one, as expected, because the code does not possess bit-flip error-correcting capabilities, and protection against bit-flip errors is entirely achieved at the level of the cat qubit.

The logical error rate per cycle is given by [4]

$$\epsilon_L = 5.6\times 10^{-2}\left(\frac{\bar{n}^{0.86}\kappa_1/\kappa_2}{1.3\times 10^{-2}}\right)^{\frac{d+1}{2}} + 2(d-1)\times 0.50e^{-2\bar{n}}, \tag{1}$$

where $\bar{n}$ is the number of photons in the cat qubit and where κ_1 is the single-photon loss rate of cat qubits and κ_2 is the two-photon stabilization rate. The logical bit-flip probability is dominated by the errors occurring during CNOT gates $p_X^{\mathrm{CX}} = 0.5e^{-2\bar{n}}$ [4, 6], thus the probability of logical bit-flip per cycle is given by $p_{X_L} = N_{\mathrm{CX}}\times p_X^{\mathrm{CX}}$, where N_{CX} is the number of CNOT gates performed during an error correction cycle.

Also, observe that this architecture does not have a conventional threshold, as the physical phase-flip rate of cat qubits increases linearly with the average photon number $\bar{n}$, eventually surpassing the phase-flip threshold of the repetition code. For a fixed value of the error parameter κ_1/κ_2, there are an optimal code distance d^* and an average photon number $\bar{n}^*$ that minimize the logical error rate. Furthermore, some of the circuits used to implement logical gates, while also fault-tolerant, do not have a threshold either [1, 3, 4].

Despite this fact, the passive suppression of bit-flip errors at the level of the low hardware layers and the use of the error-correcting code for phase-flip correction exclusively enables to reach sufficiently low logical error rates for practical applications with a competitive overhead compared to other approaches [3, 4]. Indeed, for the typical values considered, the minimal logical error rate is extremely low. For the repetition code at $\kappa_1/\kappa_2 = 10^{-4}$, for $d = 81$ and $\bar{n} = 38$ photons, the logical error rate is given by $\epsilon_L = 10^{-31}$. Thus, it is clear that the absence of a threshold will not be a limiting factor for the logical error rate. Rather, the crucial hypothesis of the architecture is the experimental validity of the exponential suppression of bit-flips. Ultimately, what will set a lower bound on the logical error probability attainable is the typical rate of physical error mechanisms that do not satisfy the error correction assumptions, just like for architectures with a theoretical threshold. In the regime of moderate noise bias $\eta \approx 10^2$ where active correction against bit-flips at the level of the code remains necessary, it was recently shown how qLDPC codes could be

* diego.ruiz@alice-bob.com

bias-tailored to improve their performance [7].

B. Current state of the experimental art and further research directions

The theoretical estimation of the performance of our codes relies on two independent hardware assumptions. The first one is about the minimal physical bit-flip error probability that can be achieved thanks to the exponential suppression of bit-flip errors with the average photon number $\bar{n} = |\alpha|^2$. Assuming for instance that $\kappa_2/2\pi = 3.18\,\mathrm{MHz}$, the average photon number $\bar{n} = 11$ corresponds to a cat qubit bit-flip time of $T_X = 13$ minutes. The second one is about the value for the ratio κ_1/κ_2, where $1/\kappa_1$ is the single-photon lifetime of the resonator hosting the cat qubit and κ_2 the two-photon stabilisation rate of the cat qubit. This ratio determines the phase-flip error models of all cat qubit operations. As a reminder, for $\kappa_1/\kappa_2 = 10^{-4}$, the $[429, 100, 22]$ code can be used to store one hundred logical qubits with logical error probability $\epsilon_L \leq 10^{-8}$, with an average photon number in the cat states $\bar{n} = 11$ (the number 758 physical cat qubits given in Table I includes the measurement ancilla qubits). We now review recent cat qubit experiments to put these numbers in perspective.

Experimental progress towards macroscopic bit-flip times. Loosely speaking, as long as the error channels of the harmonic oscillator encoding the cat qubit induce local errors in phase space, the increasing separation of the states $|\alpha\rangle$ and $|-\alpha\rangle$ with the average number of photons allows for the exponential suppression of the resulting bit-flips at the encoding level. The bit-flip rate saturates at the rate of the first physical phenomenon that induces non-local errors in the phase space. This is somewhat similar to the discrete-variable error correcting codes, like the surface code: the exponential suppression of logical errors with the code distance assumes that errors are acting locally and independently on each physical qubits. In experiments, this exponential suppression of logical errors is indeed observed until it saturates at a rate corresponding to the dominant physical error channel that induces spatially correlated errors over many qubits (in the case of [8], this is due to high-energy cosmic rays). The first experimental demonstrations of the stabilization of cat states with two-photon dissipation [9, 10] did not show any exponential bit-flip suppression. In these experiments, the two-photon exchange medium was a parametrically driven transmon-type circuit. These experiments suffered from strong parasitic interactions (stronger than the engineered two-photon dissipation) that led to non-local perturbations, and prevented any bit-flip suppression. The subsequent experiments replaced this two-photon exchange circuit with an Asymmetrically Threaded SQUID (nicknamed ATS) that removed most of these parasitic interactions, and demonstrated the exponential suppression of bit-flips, resulting in an improvement of up to 300 times the bare lifetime of the resonator [11]. In this experiment, the suppression of bit-flips saturated around 5 photons, achieving a bit-flip lifetime of $T_X = 10^{-3}\,\mathrm{s}$ (for a phase-flip rate at 5 photons of $T_Z = 5 \times 10^{-7}\,\mathrm{s}$). The saturation of the bit-flip time was attributed to strong dispersive coupling with a thermally excited transmon (used for the quantum state tomography of the cat state). A follow-up experiment confirmed that this saturation was indeed due to the transmon [12] and that by removing it, macroscopic bit-flip times of 100 seconds should be achievable. This was demonstrated in a recent experiment [13], where a bit-flip time of $T_X = 15\,\mathrm{s}$ was observed for a cat qubit with $\bar{n} = 11.3$ photons (for a corresponding phase-flip rate of $T_Z = 4.9 \times 10^{-7}\,\mathrm{s}$).

The exact physical error channel causing the bit-flip saturation at a scale of a few tens of seconds remains unclear. However, the impact of high-energy cosmic rays causing non-local errors on the superconducting chip was identified in surface code experiments as the limiting factor for the exponential suppression of logical errors [14]. The impact of such events on cat qubits is not yet clear, but the time scales involved are similar, which suggests that it might be the limiting factor.

Experimental progress for κ_1/κ_2. In order to decrease κ_1/κ_2, two complementary approaches may be used: increase κ_2 and decrease κ_1.

We first summarize recent progress to increase κ_2. Using 2D microwave resonators, the initial experiment that demonstrated the stabilization of a cat state achieved κ_1/κ_2 ratios close to 1 ($\kappa_1/2\pi = 53\,\mathrm{kHz}$, and $\kappa_2/2\pi = 40\,\mathrm{kHz}$ in Lescanne *et al.* [11]). Since this work, progress in cat qubit circuit engineering has allowed to increase this ratio by more than two orders of magnitude to achieve more recently a ratio $\kappa_1/\kappa_2 = 6.5 \times 10^{-3}$ ($\kappa_2/2\pi = 2.16\,\mathrm{MHz}$, $\kappa_1/2\pi = 14\,\mathrm{kHz}$) [15]. These circuits may be further optimized, and there is no fundamental theoretical reason to think that one cannot achieve higher values for κ_2, perhaps in the range $\kappa_2/2\pi \geq 10\,\mathrm{MHz}$. Therefore, we deem a value around $\kappa_1/\kappa_2 = 10^{-4}$ may be achieved when the platform reaches its experimental maturity.

In order to achieve lower values for κ_1, besides progress in material science or general microwave packaging, a different approach involves using a different resonator technology. This could be achieved *e.g.* by using 3D resonators whose lifetimes can reach up to 34 ms ([16], corresponding to a $\kappa_1/2\pi = 4.7\,\mathrm{Hz}$). Another approach would be to use acoustic resonators coupled to superconducting circuits (see [3] for an analysis with cat states where $\kappa_1/\kappa_2 = 10^{-5}$ is proposed with $\kappa_1/2\pi = 2.8\,\mathrm{Hz}$, $\kappa_2/2\pi = 280\,\mathrm{kHz}$). For such a change of resonator technology, the layout of Fig. 4(d) with 2D superconducting resonators would need to be adapted accordingly.

While these recent experimental works on dissipative cat qubits have characterized the bit-flip time for an idling cat qubit, it was demonstrated in [13] that the macroscopic bit-flip times were preserved while rotations

around the Z axis of the Bloch sphere were performed on the cat qubit. An important assumption that our architecture relies on is that all of the physical gates applied to the cat qubit preserve the noise bias to a sufficient level so that the bitflip correction can be avoided, which has yet to be experimentally demonstrated, *e.g.* for the two-qubit CNOT gate, although recent progress towards this goal has been made [17, 18]. Furthermore, the demonstration of robust suppression of bit-flips in a multi-cat qubit chip also needs to be done.

C. Theoretical optimization

The cat qubit architecture has also been the subject of many very recent theoretical proposals that have not yet been realized experimentally, but which are expected to potentially relax the constraint on κ_1/κ_2 by an order of magnitude. That is, to achieve figures close to those in Table I for $\kappa_1/\kappa_2 = 10^{-3}$, or alternatively, to reach a logical error rate $\epsilon_L = 10^{-13}$ with $\kappa_1/\kappa_2 = 10^{-4}$.

A first optimization is the use of asymmetric repetition codes [6], whose performance is increased thanks to the "specialization" of cat qubits. More specifically, it has been demonstrated that the more relevant ratio is κ_1^d/κ_2^a, where the indices a and d refer to the ancilla and data cat qubits of the repetition code, respectively, since κ_1^d sets the typical decoherence time of the quantum information to be protected and $1/\kappa_2^a$ sets the typical error syndrome extraction time. By designing an architecture where κ_1^d and $1/\kappa_2^a$ are minimized (even if it means increasing κ_1^a and $1/\kappa_2^d$), it is, for example, possible to maintain a logical error rate $\epsilon_L = 10^{-7}$ with a repetition code of distance $d = 11$ and $\kappa_1/\kappa_2 = 6.3 \times 10^{-4}$ for an asymmetry $\kappa_1^a/\kappa_1^d = \kappa_2^a/\kappa_2^d = 20$, instead of $\kappa_1/\kappa_2 = 10^{-4}$ for a symmetric architecture (see Figure 10 of [6]).

A second optimization is the use of squeezed cat states $|\mathcal{C}^{\pm}_{r,\alpha}\rangle \doteq S(r)|\mathcal{C}^{\pm}_{\alpha}\rangle$, where $S(r) = \exp\left[\frac{r}{2}(a^2 - a^{\dagger 2})\right]$ is the squeezing operator. It has been recently theoretically demonstrated [5, 19, 20] that the scaling of the exponential suppression of bit-flips increases with the squeezing parameter, $O(e^{-\gamma(r)\bar{n}})$, where $\gamma(0) \approx 2$ for cat states and $\gamma(r) > 2$ for squeezed cat states ($r > 0$). Consequently, the targeted bit-flip error probability can be achieved at smaller photon numbers, which allows for an identical physical phase-flip error probability for a higher value of κ_1/κ_2.

A third optimization is the use of an additional Hamiltonian confinement [21]. In this paper, a purely dissipative architecture where the cat states are stabilized by two-photon dissipation at a rate κ_2 modeled by the Lindbladian operator $D[a^2 - \alpha^2]$ is considered. It has been recently shown how the fidelity and speed of gates on cat qubits can be improved by confining the cats with degenerate Hamiltonians whose ground state corresponds to the code space of the cats [21–24]. The main difficulty with this approach is to preserve the exponential suppression of bit-flips in the presence of these confinement Hamiltonians [25].

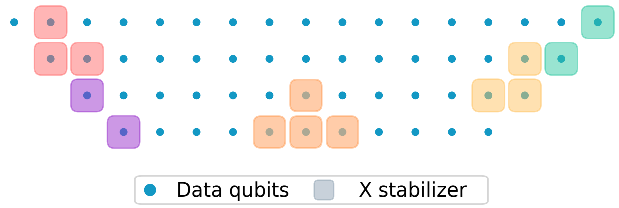

Supplementary Figure 1. Cellular automaton code without periodic boundary conditions. The number of logical qubits encoded still corresponds to the number of physical qubits in the bottom row, but the lattice is extended on both sides to enable the logical qubits on the side to have the same distance. The stabilizer which overlaps with the edges are simply truncated.

A potential final optimization comes from the use of autonomous feedback to correct first-order phase-flip errors induced during gates, which can drastically reduce the gate errors for dissipative cat qubits [26]. Furthermore, with squeezed cat states, this autonomous feedback can also be used to correct errors due to photon loss using the same process.

The study of the precise gains provided by each of these optimizations, and how they can be combined, is out of the scope of this paper.

II. CELLULAR AUTOMATON CODES

A. Removing periodic boundary conditions

The local codes were searched by imposing periodic boundary conditions on the lateral edges, in order to avoid having to consider the precise shape of the code boundaries, which depend on the shape of the stabilizers. For a practical 2D implementation, however, it is crucial to remove these periodic conditions to preserve the locality of the code. Nevertheless, it is essential to ensure the codes are made planar without compromising the parameters $[n, k, d]$. This can be done in the case of cellular automaton code as illustrated in Supplementary Figure 1 by adding data qubits on both edges, in order to preserve the support of the logical operators Z_L which previously "looped" to the opposite side of the chip. The stabilizers at the edge are then simply truncated when they exceed the grid of physical qubits. While this requires adding a few physical qubits in order to maintain the code distance, this overhead is negligible in practice for the typical regime of interest ($d \approx 10-30$ and $k \approx 100-10,000$). Indeed, for a cellular automaton code, the number of qubits added on the sides is on the order of H^2, independent of the lattice width L which allows increasing the number of logical qubits.

B. Logical error simulation details and threshold analysis

The phase-flip threshold of cellular automaton codes is lower than for the repetition codes as seen in Figure 3. To evaluate the impact of the syndrome measurement circuit depth, which is longer for cellular automaton codes, we consider in addition to the *cat qubit circuit-level* error model, two other error models, summarized in Supplementary Table 1. The first model is a *phenomenological* model (a) where each data qubit undergoes a phase-flip with probability p before each error correction round, and each syndrome is read erroneously with an error probability $q = p$. This model allows for the comparison of intrinsic features of the codes, such as the phase-flip threshold or the scaling of the logical error below threshold (*i.e.* the scaling of the exponential suppression of errors with code distance). Next, we study the codes with a *generic circuit-level* error model (b), where all code operations have a generic phase-flip error model parameterized by the operation infidelity p, assumed identical for all operations. This error model captures the effect of syndrome extraction circuits, such as the impact of the weight of the stabilizers. Finally, the *cat qubit circuit-level* error model (c) includes errors at the same locations as the generic circuit-level model, but with the precise error models of noisy operations on cat qubits [1, 3] parameterized by the relevant ratio κ_1/κ_2, where κ_1 is the single-photon loss rate of cat qubits and κ_2 is the two-photon stabilization rate. In this model, the different types of phase-flip errors do not happen with the same probability, in particular, the CNOT gate infidelity is dominated by ancilla phase-flip errors, which correspond to syndrome measurement errors.

To also evaluate the impact of the stabilizer shape, we numerically calculate the logical error probability for the cellular automaton code family characterized by the unique stabilizer shape 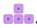, as well as the optimized codes of Table 2, and compare their performance to the repetition code family. In the asymptotic regime (p or $\kappa_1/\kappa_2 \ll 1$), the logical error probability is dominated by error configurations where there are exactly $\lfloor (d+1)/2 \rfloor$ errors. Therefore, the total error probability $p_{Z_L}^{\text{tot}}$ after d rounds of error correction cycles is fitted to the ansatz [3]

$$p_{Z_L}^{\text{tot}} = Ad(Bp)^{C\lfloor \frac{d+1}{2} \rfloor} \tag{2}$$

or

$$p_{Z_L}^{\text{tot}} = Ad(B\kappa_1/\kappa_2)^{C\lfloor \frac{d+1}{2} \rfloor}. \tag{3}$$

The fitted parameters (A, B, C) are summarized in Supplementary Table 1. We plot the logical error probability as a function of the physical noise error probability in Supplementary Figure 2 for (a) the phenomenological model with $p = q$, (b) the generic circuit-level model, and (c) the circuit-level model of cat qubits. Initially, we observe in Supplementary Figure 2 (a) that for a phenomenological model, the codes appear to have the same phase-flip threshold and the same scaling below the threshold, since the curves with the same distance are almost perfectly superimposed. Note in particular that the cellular automaton code with different stabilizer shapes in each row has the same logical error as the repetition code or single-shape cellular automaton code of identical distance. This indicates that the appropriate metric characterizing code performance is the distance, and that the weight or shape of the stabilizers does not play a significant role in the case of cellular automaton codes when the circuit measurement depth is not taken into account as in the phenomenological model. In Supplementary Figure 2(b), we see that, under generic circuit-level phase-flip noise, the phase-flip threshold of cellular automaton codes is divided by approximately a factor two. We attribute this to the fact that the syndrome measurement circuit is roughly twice deeper for these codes, as the weight-four stabilizers require four CNOT gates to be measured instead of two for the weight-two repetition code. Finally, in Supplementary Figure 2(c), we represent the logical phase-flip error as a function of κ_1/κ_2 at a fixed number of photons $\bar{n} = 11$ which exhibits similar phase-flip threshold reduction.

III. FAULT-TOLERANT OPERATIONS

A. Pauli measurements and preparations

Pauli measurements. The logical qubits can be simultaneously destructively measured in the logical Z_L basis (typically, at the end of the algorithm) by measuring all of the data cat qubits in the Z basis. The value of each of the logical Z_L operators is then obtained by multiplying all the measurement outcomes corresponding to the qubits in their support. Here, the fault-tolerance is guaranteed from the fact that only an (exponentially suppressed) bit-flip error can flip the logical measurement outcome. Similarly, the logical qubits can be simultaneously destructively measured in the logical X_L basis by measuring all of the data cat qubits in the X basis. The results of the measurement outcomes are used to infer the value of the stabilizers, which are fed to the decoder. The final measurement outcomes of the X_L operators are then deduced from the measurement outcomes of the $(m-1)$ bottom rows by flipping the measurement outcomes of the qubits where the decoder predicted a phase-flip error. Note that it is not possible to simultaneously destructively measure all of the logical qubits in different bases in a fault-tolerant manner, as the physical qubits of each logical qubit measured in the logical Z_L basis are measured in the Z basis, such that the corresponding measurement outcomes may no longer be used to infer the value of the X stabilizers in which they participate.

Alternatively, the logical Pauli operator Z_L of any logical qubit in the memory layer can be measured in a quantum non-demolition (QND) manner using the computing

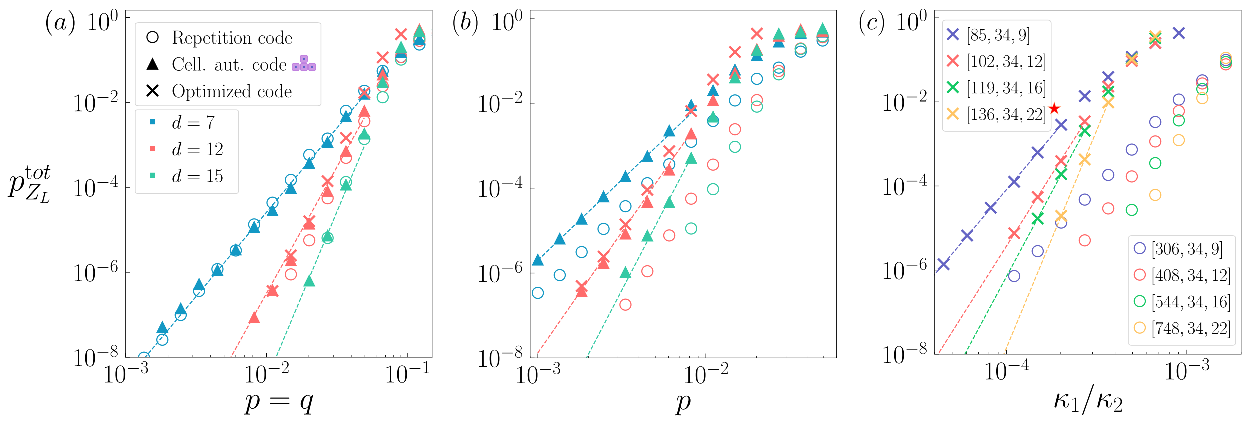

Supplementary Figure 2. Logical phase-flip error probability normalized by the number of logical qubits after d error correction cycles $p_{Z_L}^{\text{tot}}$ as a function of the physical error probability, under (a) a phenomenological error model, (b) a generic phase-flip circuit level error model, or (c) a cat qubit circuit-level error model, which are detailed in Supplementary Table 1. Repetition codes are identified by circles, cellular automaton codes by triangles, and optimized codes from Table 2 by crosses. In (a)-(b), repetition codes are compared to the cellular automaton code family to understand generic features of these codes. We observe that codes with identical distances have identical logical error probability under a phenomenological error model (a), which indicates that the repetition code and cellular automaton code have similar phase-flip thresholds. In (b), the effect of the depth of stabilizer measurement circuits becomes visible: the phase-flip threshold of weight-4 codes (cellular automaton and optimized codes) is roughly divided by a factor two compared to the weight-2 repetition codes. In (c), we compare the performance of the best codes we found (Table 2) under circuit-level noise, since these codes are the ones that will be implemented in practice. The fit in (c) is used to extrapolate the logical error rate of the $d = 22$ code to $\kappa_1/\kappa_2 = 10^{-4}$.

		(a) Phenomenological	(b) Generic circuit-level	(c) Cat qubit circuit-level
$\mathcal{P}_{\vert+\rangle}$	Z	-	p	$\bar{n}\kappa_1 T_{\text{prep}}$
	Idle	p	p	$\bar{n}\kappa_1 T_{\text{prep}}$
$\mathcal{M}_X$	$1-\mathcal{F}$	$p = q$	p	$\bar{n}\kappa_1 T_{\text{meas}}$
	Idle	-	p	$\bar{n}\kappa_1 T_{\text{meas}}$
CNOT	Z_c	-	$p/3$	$\bar{n}\kappa_1 T_{\text{CX}} + \pi^2/(64\bar{n}\kappa_2 T_{\text{CX}})$
	Z_t	-	$p/3$	$0.5\bar{n}\kappa_1 T_{\text{CX}}$
	Z_cZ_t	-	$p/3$	$0.5\bar{n}\kappa_1 T_{\text{CX}}$
	Idle	-	p	$\bar{n}\kappa_1 T_{\text{CX}}$
		$p_{Z_L}^{\text{tot}} = Ad(Bp)^{C\lfloor\frac{d+1}{2}\rfloor}$		$p_{Z_L}^{\text{tot}} = Ad(B\kappa_1/\kappa_2)^{C\lfloor\frac{d+1}{2}\rfloor}$
Repetition code	A	0.32	0.12	0.07
	B	6.2	23	486
	C	1	0.99	0.94
Cellular automaton codes	A	0.07	0.019	-
	B	8.3	53	-
	C	0.99	0.95	-
Optimized code [136, 34, 22]★	A	-	-	0.1
	B	-	-	1613
	C	-	-	0.94

Supplementary Table 1. (Top) Physical error probabilities of the operations used in the stabilizer measurement circuits. The "idle" error probability corresponds to the phase-flip error probability applied to all the qubits that are idling while the corresponding operation is performed. (a) For a phenomenological error model, the phase-flip error applied to the data corresponds to an idling error with probability p while the ancilla qubits are prepared in the $|+\rangle$ state. (b) For a generic phase-flip circuit-level noise, all of the operations have identical infidelity p. (c) The cat qubit circuit-level noise (see [1, 3] for a detailed analysis) depends on the time of the operations T_{gate}, the single-photon loss rate κ_1 and the average photon number $\bar{n}$. In our simulation, we considered an average photon number $\bar{n} = 11$ and an identical time for all operations $T_{\text{prep}} = T_{\text{meas}} = T_{\text{CX}} = 1/\kappa_2$, where κ_2 is the two-photon dissipation rate. In this case, the errors are parametrized by the unique parameter κ_1/κ_2. (Bottom) Fitting parameters for the different codes and error models. The cellular automaton codes with different distances are fitted together as they form a code family, while the optimized code is fitted independently (setting $A = 0.1$). The fitting parameter C is close to 1, indicating that the logical error probability is dominated by error configurations with exactly $\lfloor(d+1)/2\rfloor$ physical errors.

layer by performing a logical transversal CNOT between the LDPC logical qubit as control and an ancillary logical qubit as target of the computing layer prepared in $|0\rangle_L$, followed by a logical Z_L measurement of this ancillary qubit. The logical Pauli operator X_L is measured by performing a physical CNOT gate between the support of the weight-one X_L operator of any logical qubit in the memory layer as target and an ancillary physical qubit of the computing layer, prepared in a $|+\rangle$ state, as control. The ancilla is then measured in the X basis, and this procedure is repeated d times to ensure fault-tolerance. Finally, the X_L measurement value is deduced with a majority vote.

Pauli preparations. While the logical $|0\rangle_L$ and $|+\rangle_L$ states could be prepared using the QND measurement of Z_L and X_L followed by the appropriate correction in case of a -1 measurement, the standard surface code method [27] that leverages stabilizer measurements can be used without resorting to the computing layer. To prepare all of the logical qubits in an eigenstate of the logical X_L operator, each physical qubit is prepared in either $|+\rangle$ or $|-\rangle$ accordingly (a state where all logical qubits are in an eigenstate of the logical X_L operator is separable). Similarly, to prepare all of the logical qubits in an eigenstate of the logical Z_L operator, each of the corresponding physical qubits in the lowest $m-1$ rows are prepared in $|0\rangle$ or $|1\rangle$, depending on the target eigenstate, and all of the qubits of the other rows are prepared in the $|0\rangle$ state. Note that this (separable) state is an eigenstate of all the logical Z_L operators with the desired eigenvalues, but is not in the code space. Therefore, d rounds of stabilizer measurements are then performed (followed by a correction that may be tracked in software) to project the state in the code space, which concludes the state preparation. Note that, symmetrically to measurements, it is not possible to prepare eigenstates of different Pauli operators simultaneously.

B. Universal gate set with magic states

In this section, we detail how magic states, encoded in repetition codes and prepared in dedicated magic state factories, can be used as a resource to perform the corresponding gate in the memory layer. We focus on the operations needed in the case of the $|\mathrm{CC}X\rangle_L = \frac{1}{2}(|000\rangle_L + |010\rangle_L + |100\rangle_L + |111\rangle_L)$ magic state, but the schemes can directly be adapted for other magic states. The implementation is shown in Supplementary Figure 3 (a) using the logical circuit depicted in Supplementary Figure 3 (b).

The logical circuits corresponding to two of the three states of the 3-qubit Toffoli magic state are shown to cover both the case of the magic state being the control and the target of the CNOT with logical qubits in the memory layer. If the magic state is the target, the first step involves "extending" or "moving" the magic state on the qubits of the computing layer connected to the qubits of the logical qubit encoded in the memory layer. This can be done with well-known stabilizer manipulation techniques [3, 27]. Then, transversal CNOT gates are applied with the logical qubit encoded in the memory layer as control. If the magic state is the control, we use lattice surgery as presented in the circuit of Figure 4(b) with the control being the magic state, a logical repetition code as an ancilla prepared in the state $|0\rangle_L$, and the target being the logical qubit in the memory layer. A transversal logical CNOT gate is performed between the magic state and the ancilla repetition code, followed by a QND logical $X_L^a X_L^C$ measurement and a Z_L^a measurement. Pauli corrections conditioned on the logical measurement outcomes complete the gate teleportation.

IV. CHIP LAYOUT

Thanks to the fact that we are considering local codes in 2D, the physical realization of the chip is greatly simplified compared to architectures that use high-encoding rate quantum LDPC codes for standard qubits, which necessarily require long-range connectivity. Here, the codes can be implemented and operated within a single memory layer, with local stabilizers of weight 4, that is, with similar constraints as the surface code that has already been experimentally realized [8]. Note that the locality of the codes alleviates the need for long-range couplers, which are technologically challenging to realize in large-scale architectures. To operate the architecture it suffices to add a computing layer with repetition codes, which remains simpler than many existing proposals to realize gates on high-rate LDPC codes [28, 29]. A possible way to realize our two-layer architecture is to use flip-chip technology, inspired by semiconductors [30] and successfully adapted to superconducting processors [31, 32]. The two layers are manufactured separately and then joined face-to-face using indium bump-bonds, where the indium establishes a superconducting galvanic connection between the two chips. Alternatively, the technology of TSV (through silicon vias) [33] would also allow for the realization of our architecture (both sides of a chip are metallized and connectivity is established through the substrate). Figure 4(d) represents all qubits, including ancillary qubits and routing qubits, and the required connectivity to implement and operate the $[165, 34, 22]$ code (the qubits of the magic state factories of the computing layer are not shown). Note that, although some lines cross, this is not a problem here. Indeed, as the architecture is realized on two planes, a line can pass from one plane to another to avoid crossings. What matters then is the density of crossings per unit cell (for footprint-on-chip reasons), which is here constant thanks to the locality of the codes.

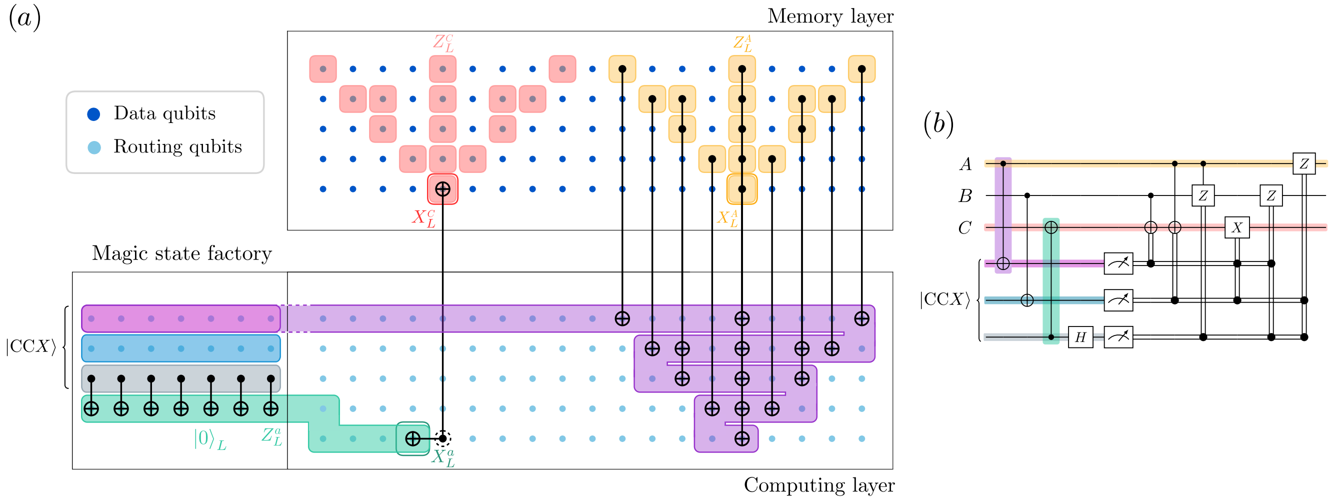

Supplementary Figure 3. Quantum circuit to perform the CCX_L gate using the $|CCX\rangle_L$ magic state. The state is prepared in a dedicated magic state factory, and quantum teleportation is used to perform the gate using the logical circuit of Figure (b). As depicted in Figure (a), the implementation requires routing qubits encoded in the repetition code. This routing circuitry can be seen as a second layer on top of the LDPC block, where CNOT are allowed between corresponding qubits in the LDPC and repetition code block. If the magic state is the target (purple), the first step involves "extending" or "moving" the magic state on the qubits of the computing layer connected to the qubits of the control logical qubit A encoded in the memory layer (yellow). Then, transversal CNOT gates are applied with the logical qubit A as control. If the magic state is the control (grey), lattice surgery is used. First, a logical $|0\rangle_L$ is prepared (green). A logical CNOT gate is performed between the control logical magic state and the logical $|0\rangle_L$. This is followed by an M_{XX} measurement between the logical $|0\rangle_L$ and the target logical qubit C (red). This process includes preparing a routing ancilla (dashed circle) in the $|+\rangle$ state, performing two CNOTs with the logical $|0\rangle_L$ and the logical X_L^C of the logical qubit C, and finally measuring the ancilla. The result is the M_{XX} measurement and the procedure is repeated d times to ensure fault-tolerance. Finally, the repetition code logical ancilla is measured in the Z basis, and Pauli corrections are applied if necessary.

[1] Jérémie Guillaud and Mazyar Mirrahimi. Repetition cat qubits for fault-tolerant quantum computation. Physical Review X, 9(4), December 2019. doi: 10.1103/physrevx.9.041053. URL https://doi.org/10.1103/physrevx.9.041053.

[2] Jérémie Guillaud and Mazyar Mirrahimi. Error rates and resource overheads of repetition cat qubits. Physical Review A, 103(4), April 2021. ISSN 2469-9934. doi: 10.1103/physreva.103.042413. URL http://dx.doi.org/10.1103/PhysRevA.103.042413.

[3] Christopher Chamberland, Kyungjoo Noh, Patricio Arrangoiz-Arriola, Earl T. Campbell, Connor T. Hann, Joseph Iverson, Harald Putterman, Thomas C. Bohdanowicz, Steven T. Flammia, Andrew Keller, Gil Refael, John Preskill, Liang Jiang, Amir H. Safavi-Naeini, Oskar Painter, and Fernando G. S. L. Brandão. Building a fault-tolerant quantum computer using concatenated cat codes. PRX Quantum, 3, 2022.

[4] Élie Gouzien, Diego Ruiz, Francois-Marie Le Régent, Jérémie Guillaud, and Nicolas Sangouard. Performance analysis of a repetition cat code architecture: Computing 256-bit elliptic curve logarithm in 9 hours with 126133 cat qubits. Physical Review Letters, 131(4), July 2023. ISSN 1079-7114. doi: 10.1103/physrevlett.131.040602. URL http://dx.doi.org/10.1103/PhysRevLett.131.040602.

[5] Qian Xu, Guo Zheng, Yu-Xin Wang, Peter Zoller, Aashish A. Clerk, and Liang Jiang. Autonomous quantum error correction and fault-tolerant quantum computation with squeezed cat qubits. npj Quantum Information, 9(1), August 2023. ISSN 2056-6387. doi: 10.1038/s41534-023-00746-0. URL http://dx.doi.org/10.1038/s41534-023-00746-0.

[6] Francois-Marie Le Régent, Camille Berdou, Zaki Leghtas, Jérémie Guillaud, and Mazyar Mirrahimi. High-performance repetition cat code using fast noisy operations. Quantum, 7:1198, December 2023. ISSN 2521-327X. doi:10.22331/q-2023-12-06-1198. URL http://dx.doi.org/10.22331/q-2023-12-06-1198.

[7] Joschka Roffe, Lawrence Z. Cohen, Armanda O. Quintavalle, Daryus Chandra, and Earl T. Campbell. Bias-tailored quantum LDPC codes. Quantum, 7: 1005, May 2023. ISSN 2521-327X. doi:10.22331/q-2023-05-15-1005. URL http://dx.doi.org/10.22331/q-2023-05-15-1005.

[8] Google Quantum AI. Suppressing quantum errors by scaling a surface code logical qubit. Nature, 614 (7949):676–681, February 2023. ISSN 1476-4687. doi: 10.1038/s41586-022-05434-1. URL http://dx.doi.org/10.1038/s41586-022-05434-1.

[9] Z. Leghtas, S. Touzard, I. M. Pop, A. Kou, B. Vlastakis, A. Petrenko, K. M. Sliwa, A. Narla, S. Shankar, M. J. Hatridge, M. Reagor, L. Frunzio, R. J. Schoelkopf,

M. Mirrahimi, and M. H. Devoret. Confining the state of light to a quantum manifold by engineered two-photon loss. Science, 347(6224):853–857, February 2015. doi:10.1126/science.aaa2085. URL https://doi.org/10.1126/science.aaa2085.

[10] S. Touzard, A. Grimm, Z. Leghtas, S. O. Mundhada, P. Reinhold, C. Axline, M. Reagor, K. Chou, J. Blumoff, K. M. Sliwa, S. Shankar, L. Frunzio, R. J. Schoelkopf, M. Mirrahimi, and M. H. Devoret. Coherent oscillations inside a quantum manifold stabilized by dissipation. Physical Review X, 8(2), April 2018. doi:10.1103/physrevx.8.021005. URL https://doi.org/10.1103/physrevx.8.021005.

[11] Raphaël Lescanne, Marius Villiers, Théau Peronnin, Alain Sarlette, Matthieu Delbecq, Benjamin Huard, Takis Kontos, Mazyar Mirrahimi, and Zaki Leghtas. Exponential suppression of bit-flips in a qubit encoded in an oscillator. Nature Physics, 16(5):509–513, March 2020. doi:10.1038/s41567-020-0824-x. URL https://doi.org/10.1038/s41567-020-0824-x.

[12] C. Berdou, A. Murani, U. Réglade, W.C. Smith, M. Villiers, J. Palomo, M. Rosticher, A. Denis, P. Morfin, M. Delbecq, T. Kontos, N. Pankratova, F. Rautschke, T. Peronnin, L.-A. Sellem, P. Rouchon, A. Sarlette, M. Mirrahimi, P. Campagne-Ibarcq, S. Jezouin, R. Lescanne, and Z. Leghtas. One hundred second bit-flip time in a two-photon dissipative oscillator. PRX Quantum, 4(2), June 2023. ISSN 2691-3399. doi:10.1103/prxquantum.4.020350. URL http://dx.doi.org/10.1103/PRXQuantum.4.020350.

[13] Ulysse Réglade, Adrien Bocquet, Ronan Gautier, Antoine Marquet, Emanuele Albertinale, Natalia Pankratova, Mattis Hallén, Felix Rautschke, Lev-Arcady Sellem, Pierre Rouchon, et al. Quantum control of a cat-qubit with bit-flip times exceeding ten seconds. arXiv preprint arXiv:2307.06617, 2023.

[14] Matt McEwen, Lara Faoro, Kunal Arya, Andrew Dunsworth, Trent Huang, Seon Kim, Brian Burkett, Austin Fowler, Frank Arute, Joseph C Bardin, et al. Resolving catastrophic error bursts from cosmic rays in large arrays of superconducting qubits. Nature Physics, 18(1):107–111, 2022.

[15] Antoine Marquet, Antoine Essig, Joachim Cohen, Nathanaël Cottet, Anil Murani, Emanuele Abertinale, Simon Dupouy, Audrey Bienfait, Théau Peronnin, Sébastien Jezouin, et al. Autoparametric resonance extending the bit-flip time of a cat qubit up to 0.3 s. arXiv preprint arXiv:2307.06761, 2023.

[16] Ofir Milul, Barkay Guttel, Uri Goldblatt, Sergey Hazanov, Lalit M. Joshi, Daniel Chausovsky, Nitzan Kahn, Engin undefinediftyürek, Fabien Lafont, and Serge Rosenblum. Superconducting cavity qubit with tens of milliseconds single-photon coherence time. PRX Quantum, 4(3), September 2023. ISSN 2691-3399. doi:10.1103/prxquantum.4.030336. URL http://dx.doi.org/10.1103/PRXQuantum.4.030336.

[17] Nathanael Cottet, Nicolas Bourdaud, Joachim Cohen, Louise Devanz, Antoine Essig, Pierre Fevrier, Antoine Gras, Jérémie Guillaud, Efe Gümüs, Mattis Hallén, et al. Towards a bias-preserving cnot gate between stabilized cat qubits (part 2). Bulletin of the American Physical Society, 2023.

[18] Sebastien Jezouin. Dissipative cat qubits for quantum computing. Bulletin of the American Physical Society, 2024.

[19] David S. Schlegel, Fabrizio Minganti, and Vincenzo Savona. Quantum error correction using squeezed schrödinger cat states. Physical Review A, 106(2), August 2022. ISSN 2469-9934. doi:10.1103/physreva.106.022431. URL http://dx.doi.org/10.1103/PhysRevA.106.022431.

[20] Timo Hillmann and Fernando Quijandría. Quantum error correction with dissipatively stabilized squeezed-cat qubits. Physical Review A, 107(3), March 2023. ISSN 2469-9934. doi:10.1103/physreva.107.032423. URL http://dx.doi.org/10.1103/PhysRevA.107.032423.

[21] Ronan Gautier, Alain Sarlette, and Mazyar Mirrahimi. Combined dissipative and hamiltonian confinement of cat qubits. PRX Quantum, 3, may 2022. doi:10.1103/PRXQuantum.3.020339. URL https://doi.org/10.1103/PRXQuantum.3.020339.

[22] Qian Xu, Joseph K. Iverson, Fernando G. S. L. Brandao, and Liang Jiang. Engineering fast bias-preserving gates on stabilized cat qubits. Phys. Rev. Research, 4:013082, Feb 2022. doi:10.1103/PhysRevResearch.4.013082. URL https://link.aps.org/doi/10.1103/PhysRevResearch.4.013082.

[23] Harald Putterman, Joseph Iverson, Qian Xu, Liang Jiang, Oskar Painter, Fernando G. S. L. Brandão, and Kyungjoo Noh. Stabilizing a bosonic qubit using colored dissipation. PRL, 128, mar 2022. URL https://doi.org/10.1103/PhysRevLett.128.110502.

[24] Diego Ruiz, Ronan Gautier, Jérémie Guillaud, and Mazyar Mirrahimi. Two-photon driven kerr quantum oscillator with multiple spectral degeneracies. Physical Review A, 107(4), April 2023. ISSN 2469-9934. doi:10.1103/physreva.107.042407. URL http://dx.doi.org/10.1103/PhysRevA.107.042407.

[25] Nicholas E. Frattini, Rodrigo G. Cortiñas, Jayameenakshi Venkatraman, Xu Xiao, Qile Su, Chan U Lei, Benjamin J. Chapman, Vidul R. Joshi, S. M. Girvin, Robert J. Schoelkopf, Shruti Puri, and Michel H. Devoret. The squeezed kerr oscillator: spectral kissing and phase-flip robustness, 2022.

[26] Ronan Gautier, Mazyar Mirrahimi, and Alain Sarlette. Designing high-fidelity zeno gates for dissipative cat qubits. PRX Quantum, 4(4), October 2023. ISSN 2691-3399. doi:10.1103/prxquantum.4.040316. URL http://dx.doi.org/10.1103/PRXQuantum.4.040316.

[27] Austin G. Fowler, Matteo Mariantoni, John M. Martinis, and Andrew N. Cleland. Surface codes: Towards practical large-scale quantum computation. Physical Review A, 86(3), sep 2012. doi:10.1103/physreva.86.032324. URL https://doi.org/10.1103/physreva.86.032324.

[28] Maxime A. Tremblay, Nicolas Delfosse, and Michael E. Beverland. Constant-overhead quantum error correction with thin planar connectivity. Physical Review Letters, 129(5), July 2022. ISSN 1079-7114. doi:10.1103/physrevlett.129.050504. URL http://dx.doi.org/10.1103/PhysRevLett.129.050504.

[29] Sergey Bravyi, Andrew W. Cross, Jay M. Gambetta, Dmitri Maslov, Patrick Rall, and Theodore J. Yoder. High-threshold and low-overhead fault-tolerant quantum memory, 2023. URL https://arxiv.org/abs/2308.07915.

[30] M. Plötner, G. Sadowski, S. Rzepka, and G. Blasek. Aspects of indium solder bumping and indium bump bonding useful for assembling cooled mosaic sensors.

Microelectronics International, 8(2):27–30, February 1991. ISSN 1356-5362. doi:10.1108/eb044447. URL http://dx.doi.org/10.1108/eb044447.

[31] D. Rosenberg, D. Kim, R. Das, D. Yost, S. Gustavsson, D. Hover, P. Krantz, A. Melville, L. Racz, G. O. Samach, S. J. Weber, F. Yan, J. L. Yoder, A. J. Kerman, and W. D. Oliver. 3d integrated superconducting qubits. npj Quantum Information, 3(1), October 2017. ISSN 2056-6387. doi:10.1038/s41534-017-0044-0. URL http://dx.doi.org/10.1038/s41534-017-0044-0.

[32] B Foxen, J Y Mutus, E Lucero, R Graff, A Megrant, Yu Chen, C Quintana, B Burkett, J Kelly, E Jeffrey, Yan Yang, Anthony Yu, K Arya, R Barends, Zijun Chen, B Chiaro, A Dunsworth, A Fowler, C Gidney, M Giustina, T Huang, P Klimov, M Neeley, C Neill, P Roushan, D Sank, A Vainsencher, J Wenner, T C White, and John M Martinis. Qubit compatible superconducting interconnects. Quantum Science and Technology, 3(1):014005, November 2017. ISSN 2058-9565. doi:10.1088/2058-9565/aa94fc. URL http://dx.doi.org/10.1088/2058-9565/aa94fc.

[33] D. R. W. Yost, M. E. Schwartz, J. Mallek, D. Rosenberg, C. Stull, J. L. Yoder, G. Calusine, M. Cook, R. Das, A. L. Day, E. B. Golden, D. K. Kim, A. Melville, B. M. Niedzielski, W. Woods, A. J. Kerman, and W. D. Oliver. Solid-state qubits integrated with superconducting through-silicon vias. npj Quantum Information, 6(1), July 2020. ISSN 2056-6387. doi:10.1038/s41534-020-00289-8. URL http://dx.doi.org/10.1038/s41534-020-00289-8.
